# A Positivity-Preserving Finite Volume Scheme for Nonequilibrium Radiation Diffusion Equations on Distorted Meshes

**DOI:** 10.3390/e24030382

**Published:** 2022-03-09

**Authors:** Di Yang, Gang Peng, Zhiming Gao

**Affiliations:** 1 Graduate School of China Academy of Engineering Physics, Beijing 100088, China; yangdi17@gscaep.ac.cn; 2 Institute of Applied Physics and Computational Mathematics, P.O. Box 8009, Beijing 100088, China; gangpengmath@foxmail.com

**Keywords:** positivity-preserving, radiation diffusion equations, finite volume scheme, distorted meshes

## Abstract

In this paper, we propose a new positivity-preserving finite volume scheme with fixed stencils for the nonequilibrium radiation diffusion equations on distorted meshes. This scheme is used to simulate the equations on meshes with both the cell-centered and cell-vertex unknowns. The cell-centered unknowns are the primary unknowns, and the element vertex unknowns are taken as the auxiliary unknowns, which can be calculated by interpolation algorithm. With the nonlinear two-point flux approximation, the interpolation algorithm is not required to be positivity-preserving. Besides, the scheme has a fixed stencil and is locally conservative. The Anderson acceleration is used for the Picard method to solve the nonlinear systems efficiently. Several numerical results are also given to illustrate the efficiency and strong positivity-preserving quality of the scheme.

## 1. Introduction

The subject capacity of entropy is huge. In thermodynamics, entropy can refer to a physical quantity that can be measured by the change of heat. In many computational problems, the nonequilibrium radiation diffusion equations play a very important role. These real physical applications include the inertial confinement fusion, Z-pinch experiments, and astrophysical problems. When the thermodynamic equilibrium between the radiation field and the material is not reached, a set of coupled radiation diffusion and material conduction equations are needed to simulate the transfer and exchange of energy. With the multiple materials, strongly nonlinear and tightly coupled with the problems, the accurate numerical method is essential to simulate the diffusion processes of these applications.

In recent years, various numerical algorithms [1,2,3] were proposed to obtain reliable numerical solutions of nonequilibrium radiation diffusion equations. The Jacobian-free Newton–Krylov method is given in [4,5] to solve the equations. In [6], the preconditioned Jacobian-free Newton–Krylov methods are considered, and it investigates in [7] a minor improvement to the operator-splitting preconditioner. The time integration methods are presented in [8,9,10], and their efficiency and accuracy are further considered in [11]. The second-order time discretization method for the coupled multidimensional flux-limited nonequilibrium radiation diffusion and material conduction equations is studied in [12]. Moreover, two different time-step control methods were given in [13]. Nevertheless, these methods cannot be used to solve the equations on distorted meshes. The radiation diffusion problems are often combined with other physical processes. For example, in Lagrangian radiation hydrodynamics, the solution is based on hydrodynamic mesh, which is often distorted by fluid motion. Therefore, the constructed numerical method should be able to simulate the radiation diffusion problem on severely distorted meshes.

Positivity-preserving is one of the key requirements for constructing the discrete scheme of nonequilibrium radiation diffusion equations. Against the background of heat transfer, if the discrete scheme does not satisfy this property, it may violate the entropy constraint of the second law of thermodynamics, and it will affect the numerical accuracy of the scheme and bring spurious oscillations. In [14,15], the positivity-preserving finite volume schemes for nonequilibrium radiation diffusion problems are constructed, and the positivity-preserving property of the schemes is also derived. With the applied interpolation method, these methods are only applicable to the problems with geometric restrictions. Moreover, the interpolation algorithms are not positivity-preserving. In [16], two finite volume element methods are presented. It was proved that one of them is monotonic, and another is positivity-preserving under some postprocessing techniques. However, these methods only can be used for the meshes with geometric restrictions. In [17], the authors propose a positivity-preserving finite point method for the nonequilibrium radiation diffusion equations. However, this method is not conservative, and it can only be used for the uniform mesh. In [18], a unified gas-kinetic scheme (UGKS) for a coupled system of radiative transport and material heat conduction with different diffusive limits was constructed, which also only considers uniform mesh.

In this paper, we propose a new positivity-preserving finite volume scheme for the nonequilibrium radiation diffusion equations based on [19,20] This numerical scheme is fixed stencil, local conservative, and positivity-preserving. The numerical scheme has the characteristics of fixed stencil, local conservative, positivity-preserving and so on. The cell-vertexes are used to define auxiliary unknowns. Therefore, vertex interpolation algorithm will be used to obtain the value of the auxiliary unknown. With the distorted meshes, the existing vertex positivity-preserving interpolation algorithms usually have significant accuracy loss. However, this scheme dose not need the interpolation method to be a positivity-preserving one. Besides, the decomposition of normal vectors on unstructured meshes is highly efficient. In practical applications, when the classical Picard iteration method solves the final nonlinear algebraic system, its convergence rate may be very slow. To speed up the convergence of nonlinear iterations, the Anderson acceleration method is used here.

The outline of this paper is as follows. In Section 2, some notations of mesh and the nonequilibrium radiation diffusion equations are presented. In Section 3, we present the construction of the new positivity-preserving finite volume scheme. Some theoretical analysis of discrete schemes is in Section 4. Then, in Section 5 we present some numerical experiments to illustrate the features of the scheme. We end our presentation in Section 6 with some conclusions.

## 2. Model and Notations

We consider a system of multimaterial nonequilibrium radiation diffusion coupled with material energy balance equation, which is defined in the domain $\Omega \in R^{2}$ with the reflection boundary conditions. The equations are written as:

(1) $$\begin{array}{c} \frac{\partial E}{\partial t}-\nabla \cdot \left(D\nabla E\right)=\sigma_{a}\left(T^{4}-E\right), \end{array}$$

(2) $$\begin{array}{c} \frac{\partial T}{\partial t}-\nabla \cdot \left(\Lambda \nabla T\right)=\sigma_{a}\left(E-T^{4}\right),\; \end{array}$$

where *E* is defined as the radiation energy density; *D* is the radiation diffusion coefficient; *T* is defined as the material temperature; Λ is the corresponding material conduction coefficient; and t is represented as time.

The energy exchange is controlled by the photon absorption cross-section:

(3) $$\sigma_{a}\left(T\right)=\frac{Z^{3}}{T^{3}},$$

where *Z* represents the atomic mass number, and its value is related to the material.

We first define the radiation diffusion coefficient without flux limiter as:

(4) $$D=\frac{1}{3\sigma_{a}}.\;\;\;$$

However, in regions with strong radiation energy gradients, the model may be unphysical, with the propagation velocity of a radiation wave front in a vacuum faster than the speed of light. To avoid this unphysical phenomenon, we add a limiting term to the diffusion coefficient and adopt such a flux-limited diffusion coefficient:

(5) $$D=\frac{1}{3\sigma_{a}+\frac{\left|\nabla E\right|}{E}}.$$

The material conduction coefficient Λ is taken as the following form:

(6) $$\Lambda =c_{0}T^{5/2},$$

where the constant $c_{0}$ is chosen to be 0.01.

Throughout this article, we employ the following notations and define the discretization of a finite volume scheme on Ω as D=(M,E,O,P), where

- M={K} denotes a family of partitions of the domain Ω into nonoverlapping mesh cells, and $\bar{\Omega }=\cup_{K\in M}\bar{K}$. For K∈M, ∂K, $h_{K}$ and |K| denote the cell boundary, diameter (the maximum distance between any two points in *K*) and measure, respectively. Besides, $h=\max_{K\in M}h_{K}$ is the mesh sizes;
- E={σ} is a finite family of disjoint edges in $\bar{\Omega }$ such that for σ∈E, σ is a line segment whose measure is defined as |σ|. Let $E^{int}=E\cap \Omega$ and $E^{ext}=E\cap \partial \Omega$. For K∈M, there exists a subset $E_{K}$ of E such that $\partial K=\cup_{\sigma \in E_{K}}\bar{\sigma }$. $n_{K,\sigma }$ denotes the unit vector normal to σ outward to *K*;
- $O=\{x_{K},K\in M\}$ is a set of points defined as cell centers, where $x_{K}\in K$;
- $P=\cup_{K\in M}P_{K}$ is also a set of point, where $P_{K}=\left\{x_{K,1},x_{K,2},\cdots ,x_{K,n_{K}}\right\}$ represents the set of vertices of cell *K*, where $x_{K,i}\;\left(1\leq i\leq n_{K}\right)$ are oriented in a counter clockwise direction. $n_{K}$ is the number of vertex for cell *K*.

Let this problem time discrete with the uniform time step Δt. $E_{K}$ and $T_{K}$ are defined as the approximate solutions of *E* and *T* at the cell center $x_{K}$ as the primary variables, respectively. The approximation of solutions *E* and *T* at the vertex point $x_{K,i}$ are defined as the auxiliary variables and denoted as $E_{K,i}$ and $T_{K,i}$.

According to the idea of finite volume framework, the above Equations (1) and (2) are integrated into each control volume *K*, and we can then obtain:

$$\begin{array}{c} \int_{K}\frac{\partial E}{\partial t}\;dx+\sum_{\sigma \in E_{K}}F_{E,K,\sigma }=\int_{K}\sigma_{a}\left(T^{4}-E\right)\;dx,\\ \int_{K}\frac{\partial T}{\partial t}\;dx+\sum_{\sigma \in E_{K}}F_{T,K,\sigma }=\int_{K}\sigma_{a}\left(E-T^{4}\right)\;dx, \end{array}$$

where $F_{E,K,\sigma }$ and $F_{T,K,\sigma }$ are the fluxes $-\int_{\sigma }\left(D\nabla E\right)\cdot n_{K,\sigma }\;ds$ and $-\int_{\sigma }\left(\Lambda \nabla T\right)\cdot n_{K,\sigma }\;ds$, respectively. $F_{E,K,\sigma }$ (resp.$F_{T,K,\sigma }$) is the one-sided flux that approximates $F_{E,K,\sigma }$ (resp.$F_{T,K,\sigma }$) using only the information of cell *K*.

## 3. Methods

In this section, we will make use of four steps to construct a new positivity-preserving finite volume scheme.

### 3.1. Construction of One-Sided Flux

In this article, we consider that the cells K∈M are star-shaped. The geometric center $x_{K}$ is the average of the cell vertices and taken as the cell center. Under the linear preserving approach [20,21], we can use the cell center $x_{K}$ and the interpolation points $P_{K}$ to obtain an approximation of the one-sided flux. The algebraic form of the flux is as follows:

(7) $$F_{E,K}=A_{E,K}\left(E_{K}I_{K}-E_{K}\right),\;F_{T,K}=A_{T,K}\left(T_{K}I_{K}-T_{K}\right),$$

where $F_{E,K}=\left(F_{E,K,\sigma },\sigma \in E_{K}\right)$ and $E_{K}=\left(E_{K,i},x_{K,i}\in P_{K}\right)$ have $n_{K}$ components, $A_{E,K}$ is an $n_{K}\times n_{K}$ cell matrix, and $I_{K}$ is a vector whose components are all 1, and its size is $n_{K}$. The $F_{T,K}$, $T_{K}$ and $A_{T,K}$ are defined similarly. Now the construction procedure of the cell matrix $A_{E,K}$ will be presented. The cell matrix $A_{T,K}$ can be obtained by the same procedure.

**Figure 1:**
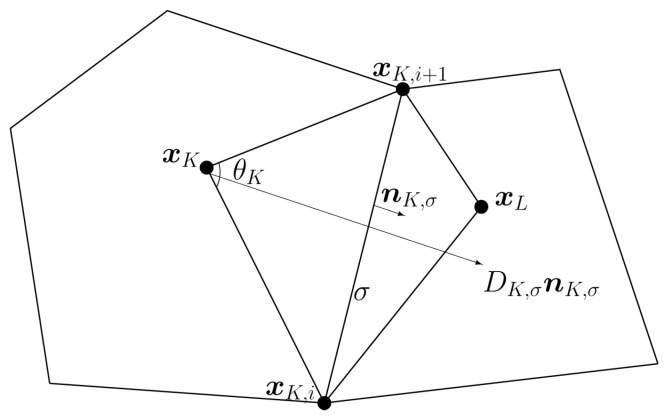
Local stencil of polyhedral mesh.

For any $\sigma \in E_{K}$, there are two endpoints $x_{K,i},x_{K,i+1}$ of σ as in Figure 1. The following decomposition can be obtained:

(8) $$\left|\sigma \right|n_{K,\sigma }=\alpha_{K,\sigma ,i}\left(x_{K,i}-x_{K}\right)+\alpha_{K,\sigma ,i+1}\left(x_{K,i+1}-x_{K}\right),$$

where

(9) $$\begin{array}{c} \begin{array}{ccc} \alpha_{K,\sigma ,i} & = & \frac{\left|\sigma \right|n_{K,\sigma }^{\prime }R\left(x_{K,i+1}-x_{K}\right)}{{\left(x_{K,i}-x_{K}\right)}^{\prime }R\left(x_{K,i+1}-x_{K}\right)},\\ \alpha_{K,\sigma ,i+1} & = & \frac{\left|\sigma \right|n_{K,\sigma }^{\prime }R\left(x_{K,i}-x_{K}\right)}{{\left(x_{K,i+1}-x_{K}\right)}^{\prime }R\left(x_{K,i}-x_{K}\right)}. \end{array} \end{array}$$

R denotes an operator that rotates a vector clockwise to its normal direction, $n_{K,\sigma }^{\prime }$ denotes the transposition of vector $n_{K,\sigma }$. Based on the decomposition (8), the linearity-preserving one-sided flux of radiation energy is given as follows:

(10) $$F_{E,K,\sigma }=D_{K,\sigma }\left[\alpha_{K,\sigma ,i}\left(E_{K}-E_{K,i}\right)+\alpha_{K,\sigma ,i+1}\left(E_{K}-E_{K,i+1}\right)\right],\;\sigma \in E_{K}.$$

The linearity-preserving one-sided flux of the material temperature *T* is defined similarly:

(11) $$F_{T,K,\sigma }=\Lambda_{K,\sigma }\left[\alpha_{K,\sigma ,i}\left(T_{K}-T_{K,i}\right)+\alpha_{K,\sigma ,i+1}\left(T_{K}-T_{K,i+1}\right)\right],\;\sigma \in E_{K}.$$

The following conditions need to be satisfied to make the discrete scheme to remain the positivity-preserving property:

(12) $$\alpha_{K,\sigma ,i}+\alpha_{K,\sigma ,i+1}>0,\;\sigma \in E^{int},$$

**Theorem** **1.** *If K is a star-shaped polygon with respect to its cell-centered $x_{K}$. With the coefficients of one-sided flux $F_{E,K,\sigma }$, $F_{T,K,\sigma }$ given by (10) and (11), the inequality (12) will be satisfied.*

**Proof.**  The star-shaped cell *K* in the mesh:

$$\begin{array}{c} {\left(x_{K,i}-x_{K}\right)}^{\prime }R\left(x_{K,i+1}-x_{K}\right)=-{\left(x_{K,i+1}-x_{K}\right)}^{\prime }R\left(x_{K,i}-x_{K}\right)=\left|\sigma \right|d_{K,\sigma }, \end{array}$$

can be obtained by considering its geometric relationship.

Then, we deduce from (9) that:

(13) $$\alpha_{K,\sigma ,i}+\alpha_{K,\sigma ,i+1}=\frac{\left|\sigma \right|n_{K,\sigma }^{\prime }R\left(x_{K,i+1}-x_{K,i}\right)}{{\left(x_{K,i}-x_{K}\right)}^{\prime }R\left(x_{K,i+1}-x_{K}\right)}=\frac{\left|\sigma \right|}{d_{K,\sigma }},$$

where $R\left(x_{K,i+1}-x_{K,i}\right)=\left|\sigma \right|n_{K,\sigma }$. Thus, the proof is completed.    □

**Remark** **1.** *We directly define $D_{K,\sigma }={\left(3\frac{z_{K}^{3}}{T_{K,\sigma }^{3}}\right)}^{-1}$ with the $T_{K,\sigma }=\frac{d_{K,\sigma }}{d_{K,\sigma }+d_{L,\sigma }}T_{K}+\frac{d_{L,\sigma }}{d_{K,\sigma }+d_{L,\sigma }}T_{L}$, to represent the radiation diffusion coefficient without flux limiter.*

*The material conduction coefficient is taken as $\Lambda_{K,\sigma }=c_{0}{\left(T_{K,\sigma }\right)}^{5/2}$. For the radiation diffusion coefficient with flux limiter, we take:*

$$\begin{array}{c} D_{K,\sigma }=\frac{1}{3\frac{z_{K}^{3}}{T_{K,\sigma }^{3}}+\frac{|\nabla E_{K,\sigma }|}{E_{K,\sigma }}}, \end{array}$$

*where $\nabla E_{K,\sigma }$ is an approximation of ∇E on triangle $▵x_{K}x_{K,i}x_{K,i+1}$ and $E_{K,\sigma }=\frac{d_{K,\sigma }}{d_{K,\sigma }+d_{L,\sigma }}E_{K}+\frac{d_{L,\sigma }}{d_{K,\sigma }+d_{L,\sigma }}E_{L}$. $\Omega_{K,\sigma }$ denotes the triangle $▵x_{K}x_{K,i}x_{K,i+1}$ (see Figure 1).*

Then, by the Green formulas:

$$\begin{array}{cc} \int_{\Omega_{K,\sigma }}\nabla Edx & =\int_{\partial \Omega_{K,\sigma }}Ends\\ \; & \approx \frac{1}{2}\left(E_{K,i}+E_{K,i+1}\right)\left|\sigma \right|n_{K,\sigma }+\frac{1}{2}\left(E_{K}+E_{K,i+1}\right)\left|x_{K,i+1}x_{K}\right|n_{x_{K,i+1}x_{K}}\\ \; & +\frac{1}{2}\left(E_{K}+E_{K,i}\right)\left|x_{K}x_{K,i}\right|n_{x_{K}x_{K,i}}\\ \; & =\frac{1}{2}\left(E_{K}-E_{K,i+1}\right)|x_{K}x_{K,i}|n_{x_{K}x_{K,i}}+\frac{1}{2}\left(E_{K}-E_{K,i}\right)\left|x_{K,i+1}x_{K}\right|n_{x_{K,i+1}x_{K}}. \end{array}$$

Here, $n_{x_{K}x_{K,i}}$ (resp.,$n_{x_{K,i+1}x_{K}}$) denotes the unit outward normal to $x_{K}x_{K,i}$ (resp., $x_{K,i+1}x_{K}$). It holds that:

$$\begin{array}{c} \frac{1}{2}|x_{K}x_{K,i}\left|\right|x_{K,i+1}x_{K}|sin\theta_{K}\nabla E_{K,\sigma }\;\;\;\;\;\;\;\;\;\;\;\;\;\;\;\;\;\;\;\;\;\;\;\;\;\;\;\;\;\;\;\;\;\;\;\;\;\;\;\;\;\;\;\;\;\;\;\;\;\;\;\;\;\;\;\;\;\;\;\;\\ =\frac{1}{2}|x_{K}x_{K,i}\left|\right|x_{K,i+1}x_{K}|\left(\frac{E_{K}-E_{K,i+1}}{|x_{K}x_{K,i+1}|}n_{x_{K}x_{K,i}}+\frac{E_{K}-E_{K,i}}{|x_{K}x_{K,i}|}n_{x_{K,i+1}x_{K}}\right). \end{array}$$

Then, we have:

$$\begin{array}{c} \nabla E_{K,\sigma }=\frac{1}{sin\theta_{K}}\left(\frac{E_{K}-E_{K,i+1}}{|x_{K}x_{K,i+1}|}n_{x_{K}x_{K,i}}+\frac{E_{K}-E_{K,i}}{|x_{K}x_{K,i}|}n_{x_{K,i+1}x_{K}}\right). \end{array}$$

### 3.2. A Unique Definition of the Facet Flux

For the interior edge $\left\{\sigma \in E_{K}\cap E_{L}\right\}$, the $F_{E,K,\sigma }$ and $F_{T,K,\sigma }$ are defined as (10) and (11). With the continuity of normal flux component, a linear combination of the one-sided fluxes is defined as follows:

(14) $${\tilde{F}}_{\vartheta ,K,\sigma }=\mu_{\vartheta ,K,\sigma }F_{\vartheta ,K,\sigma }-\mu_{\vartheta ,L,\sigma }F_{\vartheta ,L,\sigma },\;{\tilde{F}}_{\vartheta ,L,\sigma }=\mu_{\vartheta ,L,\sigma }F_{\vartheta ,L,\sigma }-\mu_{\vartheta ,K,\sigma }F_{\vartheta ,K,\sigma },$$

where $\mu_{\vartheta ,K,\sigma }$, $\mu_{\vartheta ,L,\sigma }$ are the parameters

(15) $$\mu_{\vartheta ,K,\sigma }+\mu_{\vartheta ,L,\sigma }=1,\;\vartheta =E,T.$$

Obviously, we have:

(16) $$\begin{array}{c} \begin{array}{c} {\tilde{F}}_{\vartheta ,K,\sigma }+{\tilde{F}}_{\vartheta ,L,\sigma }=0,\;\vartheta =E,T,\;\sigma \in E^{int}. \end{array} \end{array}$$

By taking (10) into the first equation of (14), we will obtain:

(17) $$\begin{array}{c} \begin{array}{c} {\tilde{F}}_{E,K,\sigma }=\left(\mu_{E,K,\sigma }D_{K,\sigma }\sum_{i=1}^{n_{K}}\alpha_{K,\sigma ,i}\right)E_{K}-\left(\mu_{E,L,\sigma }D_{L,\sigma }\sum_{i=1}^{n_{L}}\alpha_{L,\sigma ,i}\right)E_{L}\\ +\mu_{E,L,\sigma }a_{E,L,\sigma }-\mu_{E,K,\sigma }a_{E,K,\sigma }, \end{array} \end{array}$$

where:

$$\begin{array}{c} \begin{array}{c} a_{E,K,\sigma }=D_{K,\sigma }\sum_{i=1}^{n_{K}}\alpha_{K,\sigma ,i}E_{K,i},\;a_{E,L,\sigma }=D_{L,\sigma }\sum_{i=1}^{n_{L}}\alpha_{L,\sigma ,i}E_{L,i}. \end{array} \end{array}$$

Based on (15) and (17), we define:

(18) $$\begin{array}{c} \begin{array}{c} \mu_{E,K,\sigma }=\frac{|a_{E,L,\sigma }|+\epsilon }{|a_{E,K,\sigma }\left|+\right|a_{E,L,\sigma }|+2\epsilon }, \end{array} \end{array}$$

and $\mu_{E,L,\sigma }=1-\mu_{E,K,\sigma }$.

The set

(19) $$\begin{array}{c} B_{E,\sigma }=\left\{\begin{array}{c} \mu_{E,L,\sigma }a_{E,L,\sigma }-\mu_{E,K,\sigma }a_{E,K,\sigma },\;\sigma \in E_{K}\cap E_{L},\\ -a_{E,K,\sigma },\;\;\;\;\;\;\;\;\;\;\;\;\;\;\;\;\;\;\;\;\;\sigma \in E_{K}\cap E^{ext}, \end{array}\right. \end{array}$$

and

$$\begin{array}{c} \begin{array}{c} B_{E,\sigma }^{+}=\frac{|B_{E,\sigma }|+B_{E,\sigma }}{2},\;\;B_{E,\sigma }^{-}=\frac{|B_{E,\sigma }|-B_{E,\sigma }}{2}. \end{array} \end{array}$$

Then, (17) can be rewritten as:

(20) $$\begin{array}{c} \begin{array}{c} {\tilde{F}}_{E,K,\sigma }=A_{E,K,\sigma }E_{K}-A_{E,L,\sigma }E_{L}+B_{E,\sigma }^{\epsilon }, \end{array} \end{array}$$

where

(21) $$\begin{array}{c} \begin{array}{cc} A_{E,K,\sigma } & =\mu_{E,K,\sigma }D_{K,\sigma }\sum_{i=1}^{n_{K}}\alpha_{K,\sigma ,i}+\frac{B_{E,\sigma }^{+}}{E_{K}+\epsilon },\\ A_{E,L,\sigma } & =\mu_{E,L,\sigma }D_{L,\sigma }\sum_{i=1}^{n_{L}}\alpha_{L,\sigma ,i}+\frac{B_{E,\sigma }^{-}}{E_{L}+\epsilon }, \end{array} \end{array}$$

and

$$\begin{array}{c} \begin{array}{c} B_{E,\sigma }^{\epsilon }=\frac{B_{E,\sigma }^{+}\epsilon }{E_{K}+\epsilon }-\frac{B_{E,\sigma }^{-}\epsilon }{E_{L}+\epsilon }.\;\;\;\;\;\;\;\;\; \end{array} \end{array}$$

Here, ϵ denotes a positive number with the machine precision.

Finally, ignoring $B_{E,\sigma }^{\epsilon }$ in (20) and by (14), a new definition of the unique edge flux is obtained:

(22) $$\begin{array}{c} \begin{array}{cc} {\tilde{F}}_{E,K,\sigma } & =A_{E,K,\sigma }E_{K}-A_{E,L,\sigma }E_{L},\;\;\;\;\;\;\;\\ {\tilde{F}}_{E,L,\sigma } & =A_{E,L,\sigma }E_{L}-A_{E,K,\sigma }E_{K}. \end{array} \end{array}$$

For the face $\sigma \in E_{K}\cap \Gamma_{D}$, we can simply define:

$${\tilde{F}}_{E,K,\sigma }=F_{E,K,\sigma }=\left(\sum_{i=1}^{n_{K}}\alpha_{K,\sigma ,i}\right)E_{K}+B_{E,\sigma }^{+}-B_{E,\sigma }^{-},$$

where $B_{E,\sigma }^{+}$ can be treated in a way similar to the interior face, while $B_{E,\sigma }^{-}$ moves to the right of the final finite volume equations.

For the face $\sigma \in E_{K}\cap \Gamma_{N}$, we can obtain the corresponding ${\tilde{F}}_{E,K,\sigma }$ by integrating the Neumann boundary on σ.

The one-sided flux of the material temperature *T* can be defined similarly.

### 3.3. Interpolation of the Auxiliary Variables

**Figure 2:**
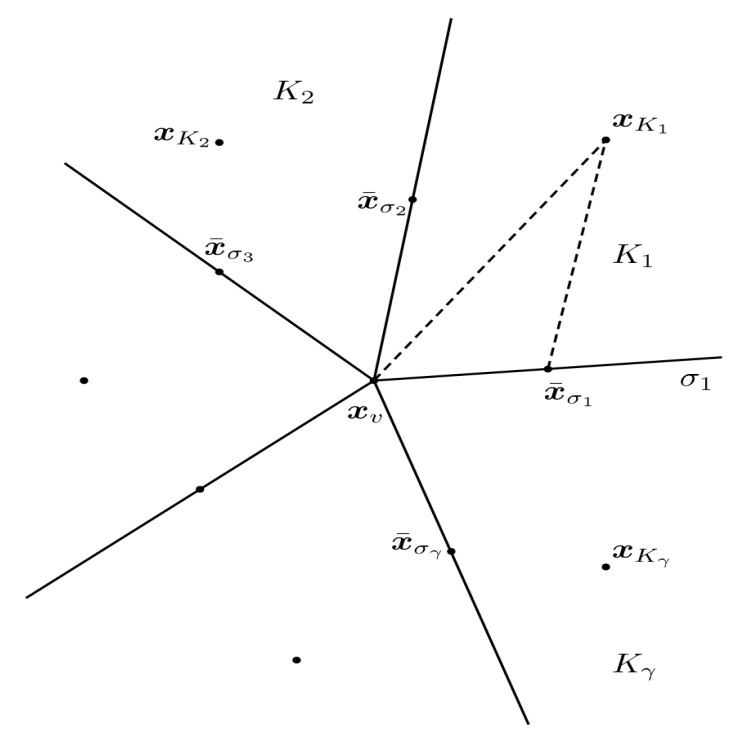
Notation and local structure around an interior mesh vertex.

To make the finite volume scheme cell-centered, we use interpolation procedure to eliminate the intermediate vertex variables in the flux expression. We express the intermediate variables as linear combinations of the primary variables. As shown in Figure 2, we might as well assume that the interior cell vertex $x_{\upsilon }$ is surrounded by cells $K_{i}$(1≤i≤γ). Let ${\bar{x}}_{\sigma_{i}}$ is the midpoint of $\sigma_{i}$, and $x_{K_{i}}$ denotes the cell center of $K_{i}$. The interior vertex $x_{\upsilon }$ is the intersection of edges $\sigma_{i}$ and $\sigma_{i+1}$. Considering the geometry of the mesh, we now that $K_{\gamma +1}=K_{1}$, $\sigma_{\gamma }=\sigma_{0}$, etc. In this section, we let $E_{\upsilon }$ represent the unknowns defined at the vertex of $x_{\upsilon }$. $D_{K_{i}}$ and $E_{K_{i}}$ are the values of *D* and *E* on the primary unknown at $x_{K_{i}}$, respectively. $S_{i,j}$ represents the area of triangle $▵x_{K_{i}}x_{\upsilon }{\bar{x}}_{\sigma_{i+j-1}}\left(j=1,2\right)$.

For i=1,2,⋯,γ and j=1,2, we define:

$$\begin{array}{c} \xi_{i,j}=\frac{D_{K_{i}}}{2S_{i,j}}{\left|{\bar{x}}_{\sigma_{i+j-1}}-x_{\upsilon }\right|}^{2},\;\;\;\;\;\;\;\;\;\;\\ {\bar{\xi }}_{i,j}=\frac{D_{K_{i}}}{2S_{i,j}}{\left({\bar{x}}_{\sigma_{i+j-1}}-x_{\upsilon }\right)}^{\prime }\left(x_{\upsilon }-x_{K_{i}}\right), \end{array}$$

and

$$\begin{array}{c} \eta_{i,j}=\frac{D_{K_{i}}}{2S_{▵x_{\upsilon }{\bar{x}}_{\sigma_{i}}{\bar{x}}_{\sigma_{i+1}}}}{\left({\bar{x}}_{\sigma_{i+1}}-{\bar{x}}_{\sigma_{i}}\right)}^{\prime }\left({\bar{x}}_{\sigma_{i+j-1}}-x_{\upsilon }\right). \end{array}$$

Then, based on the interpolation algorithm LPEW2, we obtain the following interpolation formula:

$$\begin{array}{c} E_{\upsilon }=\sum_{i=1}^{\gamma }\omega_{i}E_{K_{i}}, \end{array}$$

where

$$\begin{array}{c} \omega_{i}=\frac{{\bar{\omega }}_{i}}{\sum_{k=1}^{\gamma }{\bar{\omega }}_{k}}\;\;\mathrm{and}\;\;{\bar{\omega }}_{k}=\frac{\eta_{k-1,1}-\eta_{k,2}}{{\bar{\xi }}_{k-1,2}+{\bar{\xi }}_{k,1}}\xi_{k,1}+\frac{\eta_{k,1}-\eta_{k+1,2}}{{\bar{\xi }}_{k,2}+{\bar{\xi }}_{k+1,1}}\xi_{k,2}. \end{array}$$

The auxiliary variables of unknown *T* in flux expressions also can be computed as the above formulations.

**Remark** **2.** *With flux discrete (10) and (11), the auxiliary variables on boundary may be used. The vertex values on the boundary can be obtained using the interpolation algorithm in [20]. For the flux-limited diffusion coefficient $D_{K_{i}}$, we have the discrete ∇E on cell $K_{i}$:*

$$\begin{array}{cc} \nabla E|K_{i}\approx \frac{1}{|K_{i}|}\int_{K_{i}}\nabla Edx\; & =\frac{1}{|K_{i}|}\sum_{\sigma \in E_{K_{i}}}\int_{\sigma }En_{K_{i},\sigma }ds\\ \; & \approx \frac{1}{2|K_{i}|}\sum_{\sigma \in E_{K_{i}}}\left|\sigma \right|\left(E_{K_{i},j}+E_{K_{i},j+1}\right)n_{K_{i},\sigma }. \end{array}$$

### 3.4. The Finite Volume Scheme

To obtain the complete discretization scheme of the Equations (1) and (2), we use backward Euler to discretize time. Based on the definitions of ${\tilde{F}}_{E,K,\sigma }$ and ${\tilde{F}}_{T,K,\sigma }$, we formulate the general cell-centered positivity-preserving finite volume scheme as follows:

(23) $$\begin{array}{c} \begin{array}{c} \frac{E^{n+1}-E^{n}}{\Delta t}m\left(K\right)+\sum_{\sigma \in \varepsilon_{K}}{\tilde{F}}_{E,K,\sigma }^{n+1}=\sigma_{a,K}^{n+1}\left({\left(T_{K}^{n+1}\right)}^{4}-E_{K}^{n+1}\right)m\left(K\right),\\ \frac{T^{n+1}-T^{n}}{\Delta t}m\left(K\right)+\sum_{\sigma \in \varepsilon_{K}}{\tilde{F}}_{T,K,\sigma }^{n+1}=\sigma_{a,K}^{n+1}\left(E_{K}^{n+1}-{\left(T_{K}^{n+1}\right)}^{4}\right)m\left(K\right), \end{array} \end{array}$$

where $\sigma_{a,K}^{n+1}=\frac{z_{K}^{3}}{{\left(T_{K}^{n+1,k}\right)}^{3}}$, m(K) is the measure of cell *K*, and ${\left(T_{K}^{n+1}\right)}^{4}\approx {\left(T_{K}^{n+1,k}\right)}^{3}T_{K}^{n+1,k+1}$.

The nonlinear scheme (23) can be expressed in the following matrix form:

(24) $$\begin{array}{c} \begin{array}{c} M\left(U^{n+1}\right)U^{n+1}=F\left(U^{n}\right), \end{array} \end{array}$$

where $U^{n+1}$ denotes the vector unknowns of cell-centroid, the right-hand side vector $F(U^{n})$ is obtained by the known value, and M(U) is the coefficient matrix.

This nonlinear algebraic system can be solved by the Picard iterative method (Algorithm 1).
**Algorithm 1** Picard method.1:Choose a small positive value $\varepsilon_{non}$ and take $U^{n+1,0}=U^{n}\geq 0$.2:**for**k=1,2,...**do**3:    Solve the linear system
(25)$$\begin{array}{c} \begin{array}{c} M\left(U^{n+1,k}\right)U^{n+1,k+1}=F\left(U^{n}\right) \end{array} \end{array}$$
to obtain $U^{n+1,k+1}$.4:    Use
(26)$$\begin{array}{c} \begin{array}{c} ∥M\left(U^{n+1,k+1}\right)U^{n+1,k+1}-F\left(U^{n+1,k+1}\right)∥\leq \varepsilon_{non}∥M\left(U^{n}\right)U^{n}-F\left(U^{n}\right)∥ \end{array} \end{array}$$
as the convergence criterion.5:**end for**

To speed up the convergence of nonlinear iterations, we employ the Anderson acceleration for the Picard iteration, which is formalized as the following Algorithm 2. The parameter *m* is a fixed positive integer, which affects the efficiency of the iteration. The solution of constrained minimization problem (27) is shifted to the solution of a saddle-point problem [22].
**Algorithm 2** Anderson acceleration of Picard method.1:Give a small positive value $\varepsilon_{non}$, and define $U^{n+1,0}=U^{n}\geq 0$.2:Apply two Picard iterations (25) and set ${\tilde{U}}^{n+1,k}=U^{n+1,k}$, $\delta U^{n+1,k}={\tilde{U}}^{n+1,k}-U^{n+1,k-1}$, k=1,2.3:**for**k=2,...**do**4:    Set $m_{k}=\min\left(m,k\right)$.5:    Solve the minimization problem
(27)$$\begin{array}{c} \begin{array}{c} \min∥\sum_{i=1}^{m_{k}}\alpha_{i}\delta U^{n+1,k-m_{k}+i}∥\;\;\mathrm{with}\;\;\sum_{i=1}^{m_{k}}\alpha_{i}=1. \end{array} \end{array}$$6:    Set
$$U^{n+1,k+1}=\sum_{i=1}^{m_{k}}\alpha_{i}{\tilde{U}}^{n+1,k-m_{k}+i}.$$7:    Let $U^{n+1,k+1}:=U^{n+1,k+1}-\min\left\{U^{n+1,k+1},0\right\}e$, where e is a vector with all entries equal to 1.8:    Set $U^{n+1}=U^{n+1,k+1}$, while the criterion (26) is satisfied.9:    Solve the linear system
$$M\left(U^{n+1,k+1}\right){\tilde{U}}^{n+1,k+1}=F\left(U^{n}\right)$$
and set $\delta U^{n+1,k+1}={\tilde{U}}^{n+1,k+1}-U^{n+1,k+1}$.10:**end for**

## 4. Theoretical Results

In this section, we consider the theoretical results of the finite volume scheme. The positivity-preserving property of the scheme is first proved. Then, the compatibility of the discrete solution and discrete flux of the scheme is given. Finally, we give the existence of solution for nonlinear discrete system.

### 4.1. The Positivity-Preserving

From the construction procedure of the finite volume scheme, we have the following theorem:

**Theorem** **2.** *Assume that the initial solution vector $U^{0}\geq 0$ and the linear algebraic equations formed by Picard iteration are solved exactly. Then, we have $U^{n+1,k+1}\geq 0$ for n≥0 and k≥0.*

**Proof.**  The nonlinear discrete system (24) obtained by the positivity-preserving finite volume scheme, in which the matrix $M(U^{n+1,k})$ has the following properties:

1. All diagonal elements of matrix $M(U^{n+1,k})$ are greater than zero.
2. All off-diagonal elements of matrix $M(U^{n+1,k})$ are not less than zero.
3. The sum of each column of matrix $M(U^{n+1,k})$ is greater than zero.

Obviously, the matrix $M^{\prime }\left(U^{n+1,k}\right)$ is an M-matrix. Therefore, it is further obtained that the matrix $M^{-1}\left(U^{n+1,k}\right)$ is nonsingular. With the nonnegative vector $F(U^{n})$, one nonnegative solution $U^{n+1,k+1}$ will be obtained. □

### 4.2. The Compatibility

**Assumption** **1.** *Assume that any mesh cell K is star-shaped; that is, the ray starting from the center of cell K intersects with only one edge of the mesh. Moreover, we assume that:*

$$E\left(x\right),T\left(x\right)\in C^{2}\left(\bar{K}\right),D,\Lambda \in C^{1}\left(\bar{K}\right),\sigma_{a}\in C\left(\bar{K}\right)$$

*Obviously, some concave meshes are still star-shaped.*

**Theorem** **3.** *Suppose that an interior vertex $x_{\upsilon }$ is surrounded by cell $K_{i}$ centered at $x_{K_{i}}$, and $\omega_{E,i},\omega_{T,i}$ are the weight coefficients of the corresponding cell center $x_{K_{i}}$, γ is the number of adjacent cells of vertex $x_{\upsilon }$. Under the Assumption 1, there is a constant C independent of h, so that*

$$|E_{\upsilon }-\sum_{i=1}^{\gamma }\omega_{E,i}E_{K_{i}}|\leq Ch^{2}$$

$$|T_{\upsilon }-\sum_{i=1}^{\gamma }\omega_{T,i}T_{K_{i}}|\leq Ch^{2}$$

**Proof.**  According to the article [20], and the derivation process of vertex weight coefficient is the linearity-preserving, it is clear that the first formula in the lemma holds, and the same holds for the second formula. □

**Theorem** **4.** *Suppose that $E\left(x\right),T\left(x\right)\in C^{2}\left(\bar{\Omega }\right)$, under the Assumption 1, there is a constant C independent of h, such that:*

$$\frac{1}{\sigma }\left|{\tilde{F}}_{E,K,\sigma }-F_{E,K,\sigma }\right|\leq Ch$$

$$\frac{1}{\sigma }\left|{\tilde{F}}_{T,K,\sigma }-F_{T,K,\sigma }\right|\leq Ch$$

**Proof.**  (10) shows that:

$$F_{E,K,\sigma }=D_{K,\sigma }\left[\alpha_{K,\sigma ,i}\left(E_{K}-E_{K,i}\right)+\alpha_{K,\sigma ,i+1}\left(E_{K}-E_{K,i+1}\right)\right]+O\left(h^{2}\right)=F_{E,K,\sigma }+O\left(h^{2}\right).$$

From the Theorem 3 we known that:

$$\frac{1}{\sigma }\left|F_{E,K,\sigma }-F_{E,K,\sigma }\right|\leq Ch$$

Similarly:

$$\frac{1}{\sigma }\left|F_{E,L,\sigma }-F_{E,L,\sigma }\right|\leq Ch$$

with

$${\tilde{F}}_{E,K,\sigma }=-{\tilde{F}}_{E,L,\sigma }$$

$${\tilde{F}}_{E,K,\sigma }=\mu_{E,K,\sigma }F_{E,K,\sigma }-\mu_{E,L,\sigma }F_{E,L,\sigma }$$

$0<\mu_{E,K,\sigma },\mu_{E,L,\sigma }\leq 0,\mu_{E,K,\sigma }+\mu_{E,L,\sigma }=1$, and |σ|=O(h) then

$$\frac{1}{\sigma }\left|{\tilde{F}}_{E,K,\sigma }-F_{E,K,\sigma }\right|\leq Ch$$

Similarly, the second formula holds. □

### 4.3. The Existence of Solution

In this subsection, we only consider the existence of discrete solution for our scheme.

**Theorem** **5.** *Given $\varphi_{\alpha }\left(x\right)\geq 0$ and $g_{\alpha }\left(x\right)\geq 0,\alpha \in \left\{e,i,r\right\}$, there exists a solution $U^{n+1}\geq 0$ for the system (24).*

**Proof.**  The system (24) can be written as $U^{n+1}=M\left(U^{n+1}\right)$ and:

$$\begin{array}{c} \begin{array}{c} M\left(U^{n+1}\right)={\left(D\left(U^{n+1}\right)+\Delta tA\left(U^{n+1}\right)\right)}^{-1}F\left(U^{n}\right).\; \end{array} \end{array}$$

To prove the existence of solution for system (24), we need to prove that the map *M* has a fixed point $U^{n+1}\geq 0$. The ${\left(D\left(U^{n+1}\right)+\Delta tA\left(U^{n+1}\right)\right)}^{\prime }$ is an M-matrix. Combined with $F(U^{n})\geq 0$, we have:

$$\begin{array}{c} \begin{array}{c} \forall \;U^{n+1}\in R^{2N},U^{n+1}\geq 0,M\left(U^{n+1}\right)\geq 0.\; \end{array} \end{array}$$

From the discretization of flux (22), we know that most column sum of matrix $A(U^{n+1})$ is zero. Then, multiplying system (24) with a constant vector (1,…,1), we have:

$$\begin{array}{c} \begin{array}{c} \sum_{\alpha =e,i,r}\sum_{K\in M}{\left[D\left(U^{n+1}\right)M\left(U^{n+1}\right)\right]}_{\alpha ,K}\leq \sum_{\alpha =e,i,r}\sum_{K\in M}{\left[F\left(U^{n}\right)\right]}_{\alpha ,K}\leq C_{1}. \end{array} \end{array}$$

Further, we can obtain:

$$\begin{array}{c} \begin{array}{c} \min_{1\leq k\leq 2N}\left\{D_{k,k}\left(U^{n+1}\right)\right\}\sum_{\alpha =e,i,r}\sum_{K\in M}{\left[M\left(U^{n+1}\right)\right]}_{\alpha ,K}\leq \sum_{\alpha =e,i,r}\sum_{K\in M}{\left[D\left(U^{n+1}\right)M\left(U^{n+1}\right)\right]}_{\alpha ,K}\leq C_{1}. \end{array} \end{array}$$

Thus, we have:

$$\begin{array}{c} \begin{array}{c} \sum_{\alpha =e,i,r}\sum_{K\in M}{\left[M\left(U^{n+1}\right)\right]}_{\alpha ,K}\leq C_{0}. \end{array} \end{array}$$

Combining with $M(U^{n+1})\geq 0$, we obtain:

$$\begin{array}{c} \begin{array}{c} \;{\left[M\left(U^{n+1}\right)\right]}_{\alpha ,K}\leq C_{0},\;\forall K\in M,\;\alpha \in \left\{e,i,r\right\}. \end{array} \end{array}$$

Define a convex compact subset $C\in R^{2N}$ as follows:

$$\begin{array}{c} \begin{array}{c} C=\{U^{n}\in R^{2N}|\;0\leq T_{\alpha ,K}^{n}\leq C_{0},\;\forall K\in M,\alpha =e,i,r\} \end{array} \end{array}$$

For w∈C, we know M(w)∈C; that is, *M* maps C into itself. For each w∈C, there is only one solution for $M\left(w\right)=F\left(U^{n}\right)$. Hence, the map *M* is well-defined and M(w) is an invertible matrix, and $U^{n}=M\left(w\right)={\left(M\left(w\right)\right)}^{-1}F\left(U^{n}\right)\in C$. As $w_{k}\to w_{0}\;\left(k\to \infty \right)$ in $R^{2N}$, we know that $M\left(w_{k}\right)\to M\left(w_{0}\right)$. Since the inverse operator is continuous, we have ${\left(M\left(w_{k}\right)\right)}^{-1}\to {\left(M\left(w_{0}\right)\right)}^{-1}$. It follows that $M\left(w_{k}\right)\to M\left(w_{0}\right)$ in $R^{2N}$, so the map *M* is continuous.

By the Brouwer’s fixed point theorem [15,21], there exists a fixed point $U^{n+1}\in C$ such that $U^{n+1}=M\left(U^{n+1}\right)$. Thus, $U^{n}$ satisfies:

$$\begin{array}{c} M\left(U^{n+1}\right)U^{n+1}=F\left(U^{n}\right). \end{array}$$

□

## 5. Numerical Results

In this section, we present some numerical examples to investigate the accuracy and efficiency of the new positive-preserving finite volume scheme on distorted meshes.

The GMRES linear solver is used to solve the linear systems with stopping tolerance $1.0\times 10^{-3}$. The stopping tolerance of nonlinear iteration is taken to be $\varepsilon_{non}=1.0\times 10^{-8}$. The discrete solution errors and flux errors are investigated in the discrete $L_{2}$ norms, which are defined by the following expressions:

$$\begin{array}{c} \begin{array}{cc} \; & E_{u}={\left(\sum_{K\in M}\left|K\right|{\left(E\left(x_{K}\right)-E_{K}\right)}^{2}\right)}^{\frac{1}{2}},\\ \; & E_{q}={\left(\sum_{\sigma \in E}S_{\sigma }(\left(F_{\sigma }-F_{\sigma }^{ext}\right)/{\left|\sigma |\right)}^{2}/\sum_{\sigma \in E}S_{\sigma }\right)}^{\frac{1}{2}}, \end{array} \end{array}$$

where $S_{\sigma }$ is the measure associated with σ, $F_{\sigma }$ is the numerical flux, and the analytical flux $F_{\sigma }^{ext}$ is evaluated by the midpoint rule. Besides, we define the $L_{2}$-norm of solution to compare the numerical results on different meshes. Let:

$$\begin{array}{c} L_{2}\left(E+T\right)=\sqrt{\sum_{K\in M}{\left(E+T\right)}^{2}m\left(K\right)}. \end{array}$$

The energy conservation error is a criterion to judge the precision of the scheme. Define the total energy:

$$\begin{array}{c} E_{total}=\sum_{K\in M}\left(E+T\right)m\left(K\right). \end{array}$$

The energy conservation error is $E_{total}^{error}=\left|E_{total}^{N}-E_{total}^{0}\right|$, where $E_{total}^{0}$ is the total energy at initial time, and $E_{total}^{N}$ is the total energy at final time. Besides, the following notations are used for the numerical tests

- itn: average number of linear iterations;
- nitn: average number of nonlinear Picard iterations;
- $E_{min}$: minimal value of the numerical solution *E*;
- $T_{min}$: minimal value of the numerical solution *T*.

### 5.1. Accuracy Test

In this test, we will consider the parabolic equation on the unit square $\Omega ={\left[0,1\right]}^{2}$ as:

$$\begin{array}{c} \begin{array}{cc} \; & \frac{\partial E}{\partial t}-\nabla \cdot \left(D\nabla E\right)=f \end{array} \end{array}$$

with the full Dirichlet boundary condition. The diffusion tensor *D* is taken as:

$$\begin{array}{c} D=\left\{\begin{array}{c} \left(\begin{array}{cc} 1 & 0\\ 0 & 1 \end{array}\right),\;\;x\leq 0.5,\\ \left(\begin{array}{cc} 10 & 3\\ 3 & 1 \end{array}\right),\;x>0.5. \end{array}\right. \end{array}$$

The exact solution is chosen to be $E=tE_{1}\left(x,y\right)$:

$$\begin{array}{c} E_{1}\left(x,y\right)=\left\{\begin{array}{c} 1-2y^{2}+4xy+6x+2y,\;\;\;\;\;\;\;\;\;\;\;\;\;\;\;\;\;\;\;x\leq 0.5,\\ -2y^{2}+1.6xy-0.6x+3.2y+4.3,\;\;\;\;\;\;x>0.5. \end{array}\right. \end{array}$$

**Figure 3:**
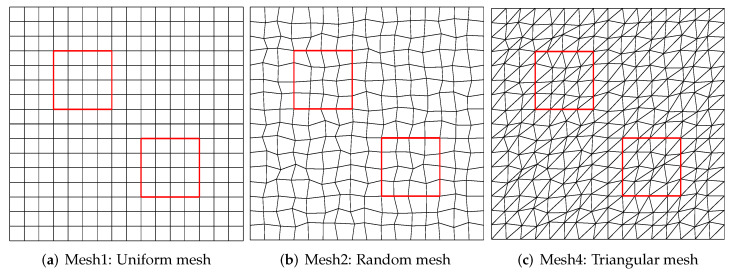
Mesh types used in numerical experiments.

**Figure 4:**
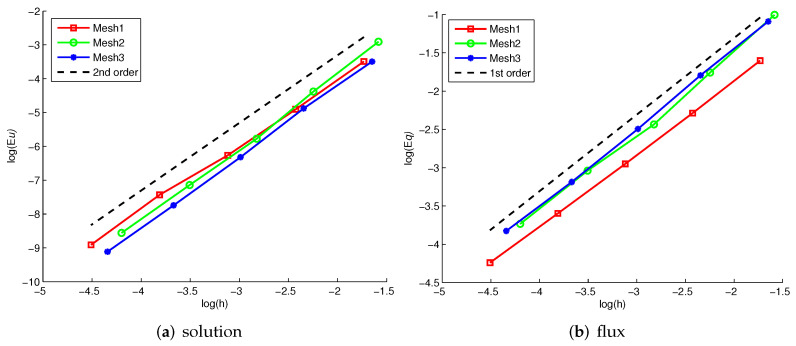
$L_{2}$ errors of solution and its flux versus mesh size *h* on various meshes.

This problem is run up to time t=0.1 with the time step $\Delta t=0.2h^{2}$. This problem is solved on three type meshes as in Figure 3, such that the nodes of unstructured mesh located on line x=0.5 are distorted only in the *y*-direction. In Figure 4, the solution and flux errors are graphically depicted as log–log plots of the discrete $L_{2}$ norm errors versus the mesh size *h*. The results demonstrate that the scheme has second (respectively first)-order convergence rate with the solution (resp. flux).

### 5.2. Results without Flux Limiter

Mousseau and Knoll present an interesting two-dimensional multimaterial test problem that has a central blast wave moving out around two square obstacles. The Equations (1) and (2) are solved on the 64×64 mesh grid as Figure 3. The background material uses Z=1, while the obstacles use Z=10. These obstacles are located at:

$$\begin{array}{c} \;\;\;\;x_{1}=0.3125\pm 0.125,\;y_{1}=0.6875\pm 0.125,\\ x_{2}=0.6875\pm 0.125,\;y_{2}=0.3125\pm 0.125. \end{array}$$

All four walls are insulated with respect to radiation diffusion and material conduction:

$$\frac{\partial E}{\partial x}{|}_{x=0}=\frac{\partial E}{\partial x}{|}_{x=1}=\frac{\partial E}{\partial x}{{|}_{y=0}=\frac{\partial E}{\partial x}|}_{y=1}=0,$$

and

$$\frac{\partial T}{\partial x}{|}_{x=0}=\frac{\partial T}{\partial x}{|}_{x=1}=\frac{\partial T}{\partial x}{{|}_{y=0}=\frac{\partial T}{\partial x}|}_{y=1}=0.$$

The initial energy distribution has a Gaussian peak near the origin:

$$E\left(x,y\right)=0.001+100\exp\left[-100(x^{2}+y^{2})\right].$$

**Figure 5:**
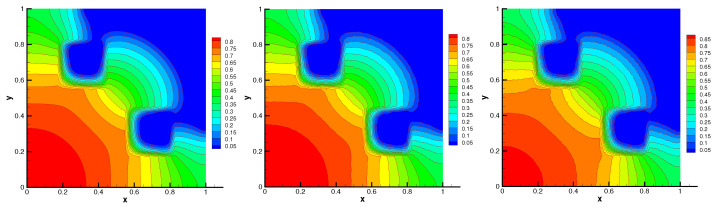
Radiation energy density on Mesh1–Mesh3 (from **left** to **right**) without flux limiter.

**Figure 6:**
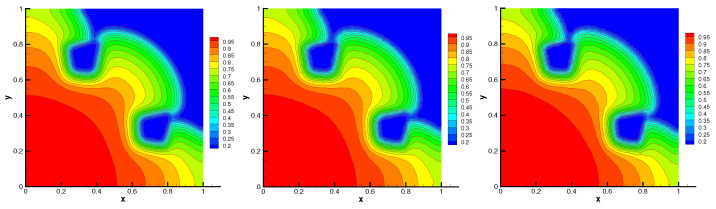
Material temperature on Mesh1–Mesh3 (from **left** to **right**) without flux limiter.

The initial material temperature is taken to be $T\left(x,y\right)=E{\left(x,y\right)}^{1/4}$. The initial radiation spreads out and flows around the obstacles. This problem is simulated with the final time t=1.5 and time step $\Delta t=5.0\times 10^{-4}$. The contour of radiation energy density and material temperature at time t=1.5 are presented in Figure 5 and Figure 6, respectively. We can see that the contours on random mesh and triangular mesh accord with that on uniform mesh. In Table 1, the minimal value and $L_{2}$-norm of numerical solution on different mesh are similar. Moreover, the minimal values of the numerical solution *E* and *T* are positive. The energy conservation error on different mesh is almost machine precision. The average number of nonlinear iterations in one time step and the average number of linear iterations per nonlinear iteration are also shown in Table 1. These numerical results indicate that our scheme is positivity-preserving, conservative, and robust in solving this problem.

The results of Anderson acceleration for the Picard iteration method are also presented in Table 2. The total number of nonlinear iterations is reduced significantly by the Anderson acceleration. The reasonable choice for this problem is m=5, since the total number of nonlinear iterative times is not observably decreasing for m=7 and m=9. From these numerical results, we know that the positivity-preserving scheme is robust and accurate in simulating this problem on distorted meshes.

### 5.3. Results with Flux Limiter

In this numerical example, we consider simulating the Equations (1) and (2) with flux limiter on the 64×64 mesh as Figure 3. The initial and boundary conditions are given as the above numerical example. This problem is run up to t=3, and the time step is taken as Δt=5.0 $\times \;10^{-4}$.

**Figure 7:**
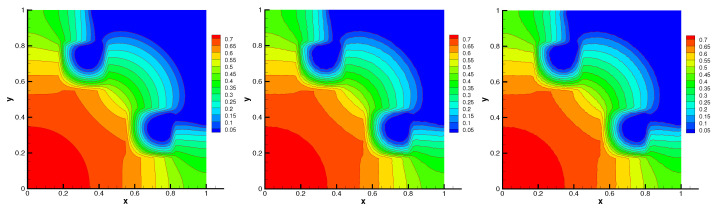
Radiation energy density on Mesh1–Mesh3 (from **left** to **right**) with flux limiter.

**Figure 8:**
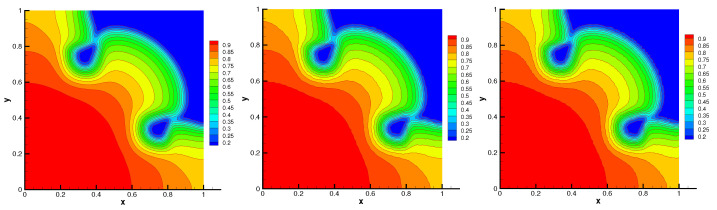
Material temperature on Mesh1–Mesh3 (from **left** to **right**) with flux limiter.

The contours of radiation energy density and material temperature are shown in Figure 7 and Figure 8, respectively. The contours on unstructured meshes also accord with that on uniform mesh. Moreover, no spurious oscillation can be seen on the distorted meshes. In Table 3, some numerical results of the radiation energy density and material temperature are presented. The energy conservation errors of this problem are almost machine-precise. The $L_{2}$-norm and minimal value of the numerical solution on distorted meshes closely approximate that on uniform mesh. The average number of nonlinear iterations and linear iterations illustrate the positivity-preserving scheme is efficient. These numerical results of the scheme are very close to the numerical solution presented in [14,15,16].

The performance of the Anderson acceleration for the Picard iteration method is also considered. In Table 4, the total number of nonlinear iterations with the different values of *m* are presented, which shows the effectiveness of Anderson acceleration method. These results illustrate that m=7 is a better choice for this test.

## 6. Discussion

We presented a positivity-preserving finite volume scheme for nonequilibrium radiation diffusion systems. The advantages of this numerical scheme are the decomposition of the normal vector and the interpolation algorithm. Moreover, it has a fixed stencil and second-order accuracy on the distorted meshes. By applying the Anderson acceleration, the number of nonlinear iterations can clearly be reduced. Besides, numerical results illustrate that this positivity-preserving scheme is an accurate method in solving the nonequilibrium radiation diffusion equations. In the future, we hope to apply the positivity-preserving finite volume scheme to more practical problems, such as three temperature radiation diffusion problem, practical multimaterial physical problems, etc.

## Figures and Tables

**Table 1:** Numerical results for problem without flux limiter.

Mesh	$E_{\min}$	$T_{\min}$	$L_{2}$ norm	nitn	itn	$E_{\mathrm{total}}^{\mathrm{error}}$
Mesh1	1.000 $\times \;10^{-3}$	0.1778	1.204	7.121	19.424	4.571 $\times \;10^{-3}$
Mesh2	9.999 $\times \;10^{-4}$	0.1778	1.207	7.391	19.567	5.435 $\times \;10^{-12}$
Mesh3	9.594 $\times \;10^{-4}$	0.1767	1.211	7.684	20.376	1.742 $\times \;10^{-12}$

**Table 2:** Numerical results of total number of nonlinear iterations.

Mesh	m=1	m=2	m=3	m=5	m=7	m=9
Mesh1	21,363	19,506	16,872	15,783	15,471	15,471
Mesh2	22,173	20,229	17,175	15,759	15,753	15,753
Mesh3	23,052	20,937	17,298	16,241	16,239	16,239

**Table 3:** Numerical results for problem with flux limiter.

Mesh	$E_{\min}$	$T_{\min}$	$L_{2}$ norm	nitn	itn	$E_{\mathrm{total}}^{\mathrm{error}}$
Mesh1	1.000 $\times \;10^{-3}$	0.1778	1.169	7.427	18.671	1.065 $\times \;10^{-12}$
Mesh2	9.911 $\times \;10^{-4}$	0.1779	1.170	7.549	18.285	6.337 $\times \;10^{-12}$
Mesh3	9.947 $\times \;10^{-4}$	0.1772	1.168	8.370	21.054	7.436 $\times \;10^{-12}$

**Table 4:** Numerical results of total number of nonlinear iterations.

Mesh	m=1	m=2	m=3	m=5	m=7	m=9
Mesh1	44,562	42,876	37,170	33,390	33,372	33,368
Mesh2	45,294	43,932	38,652	36,006	35,151	35,144
Mesh3	50,220	47,484	42,222	39,361	37,281	37,275

## Data Availability

Not applicable.
